# A Challenging Giant Dermatofibrosarcoma Protuberans on the Face

**DOI:** 10.1155/2016/5926307

**Published:** 2016-06-30

**Authors:** Gimena Castro Pérez, Cintia Arias, Paula Luna, Irene Sorín, Luis Daniel Mazzuoccolo

**Affiliations:** ^1^Dermatology Department, HIGA Eva Perón, 1650 Buenos Aires, Argentina; ^2^Dermatology Department, Hospital Aleman, 1118 Buenos Aires, Argentina; ^3^Pathology Department, HIGA Eva Perón, 1650 Buenos Aires, Argentina

## Abstract

Dermatofibrosarcoma protuberans (DFSP) is a malignant fibrohistiocytic tumor that appears exclusively on the skin. It is a low-grade malignant soft tissue tumor of subcutaneous tissues that has a propensity for local recurrence but seldom metastasizes. It may rarely occur on the head and neck accounting for less than one percent of total head and neck malignancies. We present a man with a giant DFSP on the face. Oncological, functional, and aesthetic aspects are set forth.

## 1. Introduction

Dermatofibrosarcoma protuberans (DFSP) is a low-grade malignant tumor that represents the most frequent skin sarcoma. A low percentage is located in head and neck [1, 2]; it grows at a slow pace, and it has low chance of metastasis but high level of recurrence [2]. Surgical treatment is the gold standard, but adjuvant therapy is needed in special cases. Close follow-up is imperative to detect frequent local recurrence.

## 2. Case Report

**Figure 1:**
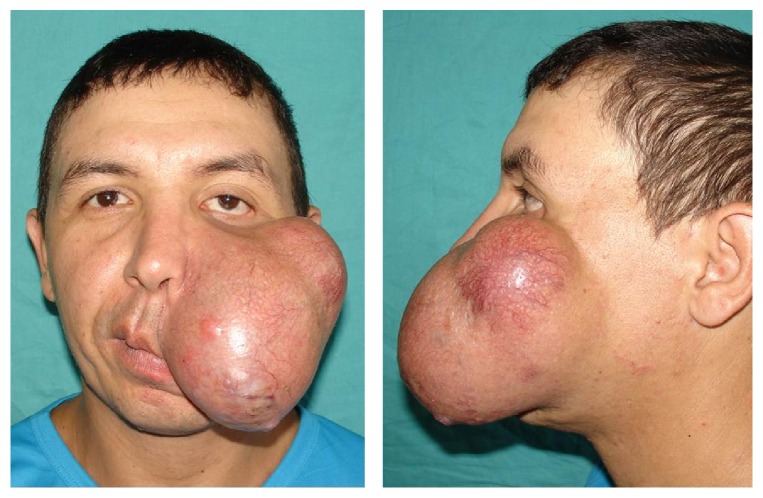
A dome-shaped, skin-colored, with smooth surface, firm tumor furrowed by telangiectasias.

A thirty-year-old male patient, previously healthy, presented to Department of Dermatology with an oval, slow growing, indolent, and disfiguring tumor on the left side of the face. The lesion had begun as an asymptomatic skin-colored, small nodule, two years before, gradually increasing until it got an enormous size of 10 × 6 cm (Figure 1).

Physical examination revealed a painless, dome-shaped, skin-colored, firm tumor, furrowed by telangiectasias that caused ectropion and mouth deviation. There were no clinical signs of oral or nasal mucosa involvement as well as no cervical or submaxillary lymphadenopathy; teeth showed no signs of disease.

A computed tomographic 3D scan of head and neck was performed, showing a vascularized tumor, with hypointense signal in T1 and hyperintense signal in T2 and a heterogeneous reinforcement after intravenous contrast administration. Tumor seemed to erode the underlying jawbone. Mucosal compromise or lymphadenomegaly were not seen.

**Figure 2:**
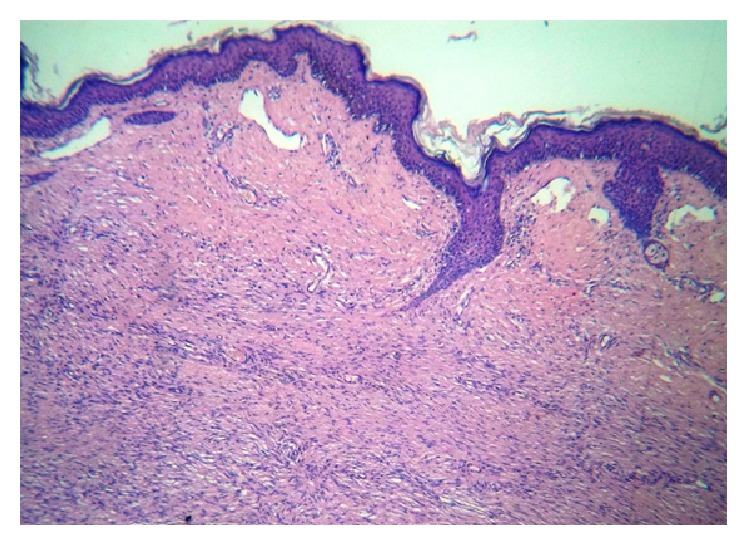
H&E panoramic view. Cutaneous section showing a dermal cell proliferation that leaves a free Grenz zone.

**Figure 3:**
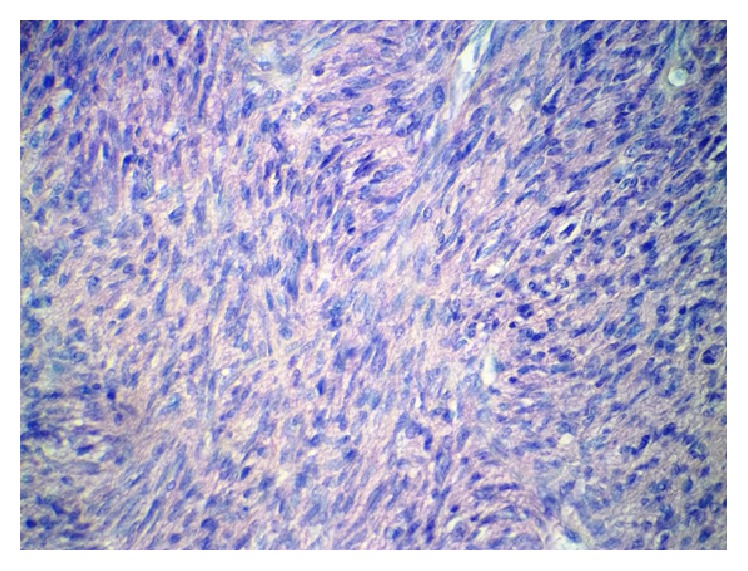
H&E 40x. Dermal infiltrate with low mitotic rate. Spindle cells mimicking a storiform pattern.

**Figure 4:**
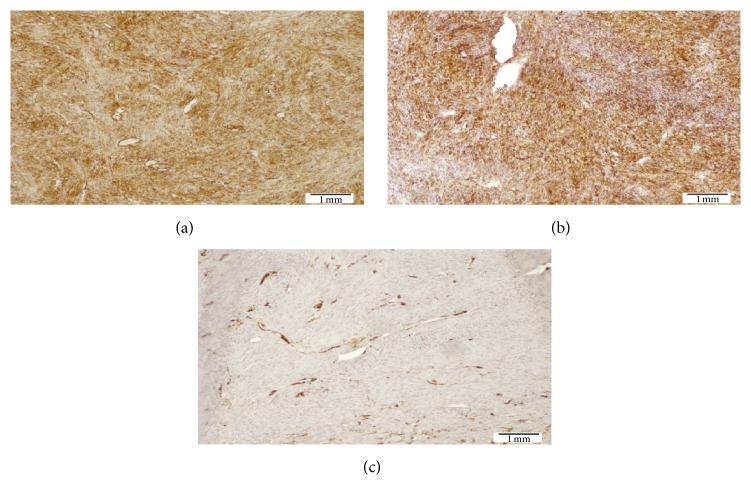
Immunohistochemistry demonstrated diffusely positive staining for CD34 (a) and vimentin (b) and negative staining for smooth muscle actin (c) consistent with DFSP.

Incisional biopsy specimen showed a mesenchymal neoplasm of spindle cells leaving a Grenz zone and mimicking a storiform pattern with scarce mitosis in the deeper layers (Figures 2 and 3). Necrosis, lymphovascular embolization, perineural invasion, or vascular proliferation was not seen. Immunohistochemical staining demonstrated S100 (−), CD99 (−), Bcl2 (−), panCK (−), Mart1 (−), desmin (−), EMA (−), cytokeratin 5/6 (−), CD68 (−), CD56 (−), HMB45 (−), CD31 (−), smooth muscle actin (−), vimentin (+), PDGFR-B (+), and CD34 (++) strongly positive. Staining for Ki 67/MIB 1 showed 11.97% positive cells (Figure 4). A diagnosis of DFSP was made.

Due to the lack of access to the gold standard treatment of Mohs micrographic surgery, the patient underwent wide surgical resection (WSR) of the tumor with a margin of 2 cm where possible. Where there was no possibility for such margins, resection was extended to muscular, aponeurotic, and periosteal planes. Special care was taken to preserve left eye, parotid gland, and facial nerve.

**Figure 5:**
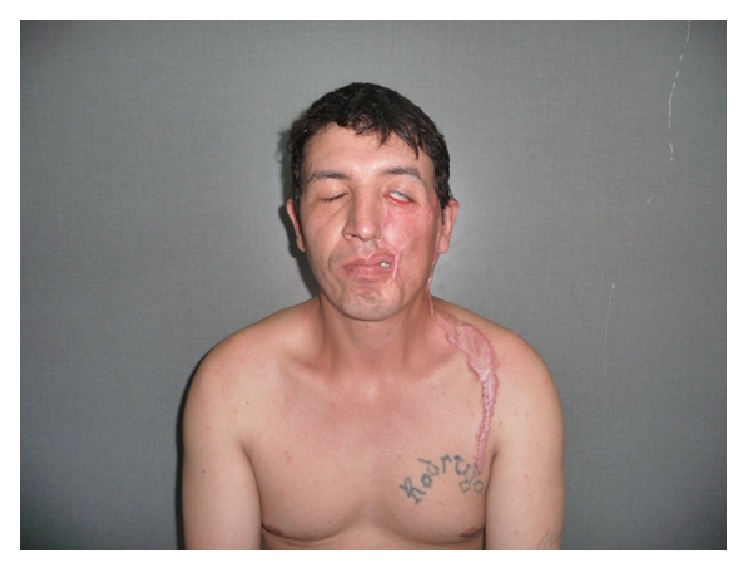
Follow-up at 36 months showing no signs of recurrence and marked disfigurement.

Histopathological examination of the surgical specimen confirmed negative margins and no periosteal compromise; in several areas, margins were informed as less than 1 cm. A pectoralis major myocutaneous flap was the therapeutic choice to repair the enormous defect left by the removal. Recovery was uneventful and the patient was discharged after seven days. Due to the reduced margins obtained in order to keep the left eye, adjuvant treatment was indicated. Despite being informed about the high risk of recurrence, the patient refused to receive any further therapy. Neither radiotherapy nor immunotherapy was done. For this reason, his treatment was considered suboptimal so a periodical screening for tumor relapse was indicated. Trimestral physical examination and CT scan showed no signs of recurrence or distant metastasis after 36-month follow-up (Figure 5).

## 3. Discussion

The first mentions of this tumor dating from 1924 described a rare low-grade soft tissue sarcoma [3]. DFSP affects adults in their third or fourth decade of life and, exceptionally, children. The trunk and extremities are the more commonly affected sites while compromise of head and neck is infrequent [4, 5].

Its annual incidence is between 0.8 and 4.5 per million and represents the most common skin sarcoma [4, 6, 7].

In first stages, it manifests as an indurated nodule that later evolves to a violaceous to red-brown irregular plaque or tumor of several centimeters in diameter fixed to dermis. It is usually asymptomatic and telangiectasias can be seen in surface. In our case, the clinical characteristics were atypical because of the tumor's smooth surface and hemispheric shape. Because of that, a DFSP diagnosis was not given on the first approach. DFSP seldom metastasizes, either to regional lymph nodes or to distant lymph nodes. However, it is locally aggressive and highly recurrent [2, 4, 8, 9].

Histological features show a spared epidermis with cellular proliferation in dermis and honeycomb pattern of infiltration into the subcutis. This proliferation is composed of spindle cells with minimal pleomorphism and low mitotic rate [4, 10]. Fibrosarcomatous transformation has been reported in the literature, sometimes associated with more aggressive behavior [2, 11]. Immunohistochemical staining shows positivity for CD34 and vimentin [12, 13]. In our case, the histopathological study showed a spindle cell proliferation that mimics storiform pattern and spared the epidermis and the superficial dermis. No vascular proliferation in staghorn pattern was seen. Immunohistochemical features were consistent with DFSP as CD34 and vimentin were diffusely positive.

The treatment of DFSP is essentially surgical [4, 9]. WSR of the lesion with 2-3 cm of surrounding healthy tissue is still the first-line therapy. Inadequate surgical margins lead to high recurrence rates [7, 9]. Even more, a recurrence rate of up to 20% with 3 cm surgical margins has been described [14]. Tumors occurring in head and neck are challenging because of the risk of disfigurement and functional impairment. That is why Mohs micrographic surgery (MMS) seems to be a better option for DFSP, especially in tumors of the head [2, 7]. It has lower rates of recurrence than WSR [15]. The possibility of multidirectional invasion, size, and location of the tumor and cosmetic issues determine the best surgical approach. In our country, there are few medical centers where Mohs surgery is performed. Our patient had no access to them. That is why WSR plus adjuvant therapy seemed to be the best available option for him.

As DFSP located on the extremities and especially on the head and neck have historically had much higher rates of recurrence than those on the trunk, primarily because it can be difficult, if not impossible, to achieve wide margins in these anatomical locations, it is imperative to design an individualized surgical approach in each patient. Surgery and adjuvant therapy recommendations are necessary for these unusual situations.

Imatinib mesylate is the adjuvant therapy approved for the treatment of unresectable, recurrent, or metastatic DFSP in adult patients [4, 9, 16]. Imatinib inhibits the platelet-derived growth factor receptor tyrosine kinase. Most DFSP show a t(17;22) chromosome translocation. This translocation is involved in pathogenesis of this tumor, with an upregulation of the platelet-derived growth factor B pathway, resulting in uncontrolled cell division. The presence of this t(17;22) chromosome translocation seems to be associated with the response to imatinib [16, 17].

Local recurrences of DFSP are frequent. Half of them are seen in the first year after surgery, but they can also occur more than five years after treatment. Head and neck is the location with more incidence of local recurrence [7, 15, 18]. This fact justifies the long term follow-up of these patients. In spite of the fact that our patient was not able to access MMS and did not agree on imatinib adjuvant therapy, he has not shown recurrence at 3-year follow-up. The chance of recurrence, the vital organs functionality, the availability of MMS, and patient preferences should be weighed up.

Our case is unique in several aspects. It was a giant, dome-shaped, and well delimitated DFSP over the face and although he was not able to receive gold standard treatment with MMS or adjuvant imatinib, no local recurrence or distant metastasis was seen at 36-month follow-up after surgical resection with narrow margins raising the issue that probably there are other prognostic factors, besides the margins, to predict recurrence.
